# Maternal plasma vitamin D levels and associated determinants in late pregnancy in Harare, Zimbabwe: a cross-sectional study

**DOI:** 10.1186/s12884-019-2362-z

**Published:** 2019-06-28

**Authors:** Raylton P. Chikwati, Cuthbert Musarurwa, Kerina Duri, Kudakwashe Mhandire, Tracy Snyman, Jaya A. George

**Affiliations:** 1 Department of Chemical Pathology, University of Zimbabwe, College of Health Sciences, P.O. Box A178, Avondale, Harare, Zimbabwe; 2 Department of Immunology, University of Zimbabwe, College of Health Sciences, P.O. Box A178, Avondale, Harare, Zimbabwe; 3 Department of Chemical Pathology, National Health Laboratory Service and University of the Witwatersrand, Parktown, Johannesburg, South Africa

**Keywords:** Vitamin D, Pregnancy, Cohort, HIV status, Harare,Zimbabwe

## Abstract

**Background:**

The importance of vitamin D in bone health and calcium homeostasis has been well documented. However, emerging evidence supports the role of vitamin D beyond its recognised traditional roles. In pregnancy, vitamin D levels are crucial in sustaining both the maternal stores and optimal growth of the foetus. In Southern Africa, there is paucity of data on vitamin D in pregnancy and related outcomes. To expand this body of knowledge, we assessed vitamin D levels in late pregnancy and (if any) associated maternal determinants in Harare, Zimbabwe.

**Methods:**

Study participants comprised of 138 pregnant Zimbabwean women in their third trimester. These were stratified by HIV status; sampling median (IQR) gestation for HIV negative study participants was 34 weeks (26–41) and 31 weeks (20–40) in the HIV positive participants.

Maternal plasma 25 hydroxyvitamin (OH) Dlevels were measured using the ClinPrepHigh Pressure Liquid Chromatography (HPLC) kit. Statistical analysis was carried out using the STATA statistical package version 13. A *p*-value of < 0.05was considered to be statistically significant.

**Results:**

HIV infected participants had significantly higher mean 25 (OH) D concentration (112 ± 33.4 nmol/L) compared to the HIV uninfected (100 ± 27.1 nmol/L), *p* = 0.032.Participants whose samples were collected during summer had higher maternal 25 (OH) D levels than those cART duration and maternal 25 (OH) D levels (*p* = 0.031, Spearman correlation =0.28).

**Conclusions:**

Our findings show high mean levels of maternal 25 (OH) D in late pregnancy in our setting and in the absence of vitamin D supplementation. Both HIV infection and season are significant determinants of maternal vitamin D levels. Summer season is associated with higher maternal plasma 25 (OH) D levels. HIV infection is associated with increased maternal vitamin D levels. Prolonged use of cART, Tenolam E is associated with improved maternal 25(OH) D levels.

## Background

The traditional roles of vitamin D [25(OH)D] in calcium and phosphorous metabolism have been well characterised. Growing evidence supports the non-traditional roles of 25(OH) D in various conditions and diseases that include infectious diseases [1–4], autoimmune diseases [5],cardiovascular diseases [6, 7], metabolic disorders [8], cancer [9] and all-cause mortality [4].

Globally, there is growing literature on suboptimal vitamin D levels (< 80 nmol/L) and associations with disease outcomes in different populations. The highest prevalence of suboptimal vitamin D levels has been reported in pregnancy [3, 10–12] and lactation [13], exclusively breastfed infants [14], human immunodeficiency virus (HIV) infected individuals [15–18] and dark skinned individuals [19, 20]. In a recent meta-analysis, Saraf and colleaguesreported that globally, 54% of pregnant women had suboptimal vitamin D levels, qualifying vitamin D status as an emerging public health problem [21].

In pregnancy, the adequate provision of vitamin D is necessary for sustainable maternal stores and for optimal foetal development [22]. Gestational concentrations of active vitamin D 1,25(OH)2D increase by 50–100% over the non-pregnant state [23]. This is due to an uncoupling mechanism on the physiological Parathyroid Hormone (PTH) role of activating vitamin D during pregnancy. As such, it is thought that the active vitamin D metabolite, 25(OH) D levels decrease to compensate this physiological change.

Many groups have reported that vitamin D deficiency is widespread among HIV-infected.

persons, with prevalence estimates ranging from 29 to 89% in European and US HIV-infected populations [17]. Only two studies within Sub-Saharan Africa have documented hypovitaminosis D in HIV positive women. One study reported 31.8% vitamin D insufficiency [25 (OH)D < 80 nmol/L] among Batswana HIV positive women at delivery who were receiving Highly Active Antiretroviral Therapy (HAART) [3]. The other study reported 39% vitamin D insufficiency among HAART naïve Tanzanian women between 12 and 27 weeks of pregnancy [4].

Furthermore, fewer studies have been also been done on healthy pregnancy women in Africa. One study was amongst Tanzanians reporting 1% vitamin deficiency (< 50 nmol/L) and 2% insufficiency (< 75 nmol/L) [24]. In a Kenyan population, Toko et al. reported that at 26 weeks gestation, the women had 51 and 21%, vitamin D insufficiency and deficiency respectively [25]. The wide discordance of these results therefore warrants further comparative studies.

To the best of our knowledge only four studies have reported serum vitamin D status on Zimbabweans. However none of these have focused on pregnancy and pregnancy related outcomes. Furthermore there is paucity of data on vitamin D status in HIV-infected populations from low income settings. We carried out a cross sectional study that estimated vitamin D status of pregnant women and assessed maternal determinants of serum vitamin D status.

## Methods

### Study setting and design

In this comparative cross sectional study, we assayed plasma 25(OH) D concentrations on archived maternal plasma samples originating from an ongoing prospective cohort study, The University of Zimbabwe Birth Cohort Study (UZBCS), which began recruitment in February 2016. Briefly, the main cohort study randomly enrolled consenting pregnant women from four primary healthcare clinics in high density suburbs of Harare, Zimbabwe. At enrolment, the study participants completed a structured questionnaire and provided blood samples. From these we identified 210 participants enrolled into the main study within the period February 2016 and August 2016. From these, 69 HIV positive and 69 HIV negative participants were randomly selected for the present study.

### Participants

Study participants consisted of randomly sampled consenting pregnant women aged 18–49 years who planned to stay in Harare for at least 1 year post delivery. Systematic sampling was used to recruit participants from a sampling frame of 210 participants (105 HIV negative, 105 HIV positive) enrolled from February 2016 to August 2016. The sampling was done by arranging the study participants according to a date based sequence from sample collection. The selection was done by removing two participants after every 10.

We excluded pregnant women with self-reported kidney disease, liver disease, gut or malabsorption disorders, and granulomatous disease and on vitamin D and/or calcium supplements. Data on socio-demographics, general maternal health, maternal medical history and anthropometric measurements was abstracted from the cohort records. We also obtained data on potential determinants of vitamin D status.

The sample size was calculated to ensure a statistical power of 80% and a probability of less than 0.05 for detecting significant differences between maternal 25 (OH) D levels and associated covariates. For the calculations, a vitamin D insufficiency prevalence of 31.8% was obtained from a study in Batswana HIV positive women at delivery (3) and 11% vitamin D insufficiency in healthy pregnant women from the NHANES study [26]. The following formula was used for calculating the adequate sample size; n = Z^2^P (1 − P)/d^2^ Where n is the sample size, Z is the statistic corresponding to level of confidence, P is expected prevalence and d is precision (corresponding to effect size). From a minimum calculated sample size of 124 participants, we recruited 137 after adding a 10% attrition factor to anticipated difficulties in assays, from which 10 were excluded from the final analysis due to technical problems during the HPLC assays. These consisted of 5 HIV negative and 5 HIV positive women.

### Laboratory methods

Plasma 25(OH) D concentrations were measured at the National Health Laboratory Services laboratory in Johannesburg, South Africa. Plasma 25(OH) D was quantified using the ClinRepHigh Pressure Liquid Chromatography (HPLC) kit (Recipe, Munchen, Germany). This method is able to resolve 25 (OH) D2 from 25 (OH) D3 using a photodiode array detector (PDA). The analytical column used for the present study consisted of octadecylsilane molecules(C18) bonded to silica particles as the stationary phase. The mobile phase used was highly polar consisting of a dilute organic solvent. A test solution was run at least twice to monitor the adequacy of the retention time and resolution of the peaks and that the baseline had stabilised. The working standard curve was used to calculate the unknown 25(OH) D2/D3 in the study samples. The standards and internal controls were prepared from commercially lyophilised pooled serum samples traceable to the National Institute of Standards and Technology Standard Reference Material 972, [25 (OH) D NIST standards. Manual integration was done on the chromatograms to better peak resolution. Quality control was also monitored to ensure conformance of each test run.

The intra-assay variation of the method ranged from 0.9–4.9% and the inter-assay variation ranged from 3.0–4.9%. The laboratory participates in a vitamin D external quality assurance scheme (DEQAS). The limit of detection for 25(OH)D3 and 25(OH)D2 was 2.5 nmol/L and 7.5 nmol/L respectively. Any 25(OH)D2 below the limit of detection was assigned a value of zero. Definitions on vitamin D status was as follows: sufficiency (80-100 nmol/L), insufficiency (< 80 nmol/L) and deficiency< 50 nmol/L [27, 28].

### Statistical analysis

Statistical analysis was carried out using the STATA statistical software package version 13 (StataCorp LP, College Station, TX, USA). The data is presented as median (IQR) if non- parametric or mean (± SD) for parametric data. The Mann-Whitney and Kruskal-Wallis were used for non-parametric comparisons. Chi square tests were used to compare categorical variables. Continuous variables were compared by the Spearman’s Correlation. Multivariate linear regression analysis was performed to determine the association between covariates and 25(OH) D levels. We used a stepwise regression approach through which all candidate variables to 25 (OH) D levels in the model were checked to see if their significance was reduced below the tolerance level of 0.1 (Table 3). All non-significant variables (*p* > 0.1) found in the model were removed.

## Results

### Study population

A total of 137 participants of whom 69 were HIV infected was randomly selected forenrolment. 25 (OH) D concentrations were available for 127 (92%) participants. The clinico-demographic characteristics of these are presented in Table 1.

**Table 1:** Participant Clinico-demographics

Characteristic	HIV negative *n* = 63	HIV positive *n* = 64	*p*-Value
Age, y (mean, SD)	26.6 (5.8)	30.3 (6.2)	< 0.001
Weight, kg (median, IQR)	68 (51–109)	68.5 (48–109)	0.725
Gestational age, wk. (median, IQR)	34 (26–41)	31 (20–40)	< 0.001
Blood Pressure, mmHg			
Systolic, median (IQR)	108 (87–153)	110 (93–138)	0.282
Diastolic, median (IQR)	67 (28–97)	69 (28–92)	0.727
Monthly income, USD	268 (40–1000)	240 (0–850)	0.777
Marital Status, *n* (%)			
Married	58 (92.1%)	43 (67.2%)	< 0.001
Single	0 (0.0%)	3 (4.7%)	0.010
Cohabitating	5 (7.9%)	16 (25.0%)	
Divorced/Separated	0 (0.0%)	2 (3.1%)	
Season of Sampling *n*, (%)			
Summer (Dec-Feb)	9 (14.3%)	8 (12.5%)	0.766
Autumn (Mar-May)	38 (60.3%)	38 (59.4%)	0.918
Winter (June-Aug)	16 (5.4%)	18 (28.1%)	0.731

The HIV infected participants were significantly older than the HIV- uninfected (*p* <  0.001).

On the other hand, the HIV uninfected had significantly more advanced pregnancy with a median (IQR) of 34(26–41) weeks (*p* <  0.001). There were no significant differences in weight, blood pressure, monthly income and season of sampling between the two groups (Table 1).

### Maternal plasma 25 (OH) D concentrations

The overall mean for maternal plasma 25 (OH) D concentration was 106 nmol/L (± 30.9) with 103 (79.5%) participants classified as vitamin D sufficient (≥ 80 nmol/L), 24 (18.9%) as insufficient (50.0–79.9 nmol/L) and 2 (1.6%) as deficient (< 50 nmol/L).

**Fig. 1:**
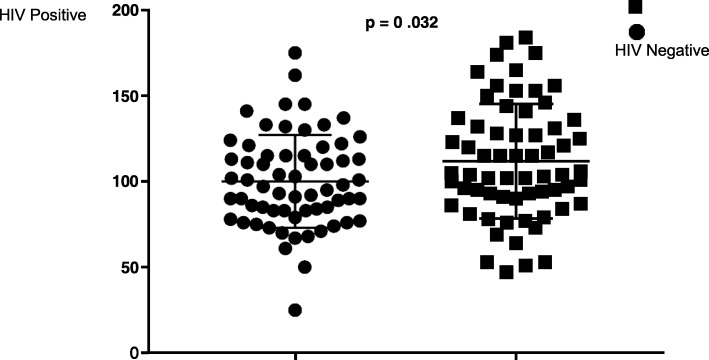
Maternal 25 (OH) D concentrations by HIV status

There was a significant difference in mean plasma 25 (OH) D between HIV negative participants, 100 nmol/L (± 27.1) and HIV positive participants, 112 nmol/L (± 33.4) (*p* = 0.032) (Fig. 1). Although mean plasma vitamin D concentration was significantly higher in the HIV infected women, there were no significant differences in the proportions of participants in each of the three pre-defined strata of vitamin D status (Table 2).

**Table 2:** Sub-categories of maternal 25 (OH) D concentrations [27, 28]

	HIV Negative *n* = 63	HIV Positive *n* = 64	*P* value
Vitamin D status, (*n*,%)			
Sufficient:	48 (76.2%)	53 (82.8%)	0.571
25(OH)D ≥ 80 nmol/LInsufficient:	14 (22.2%)	10 (15.6%)	0.564
25(OH)D50.0–79.9 nmol/LDeficient:25(OH) D < 50 nmol/L	1 (1.6%)	1 (1.6%)	0.943

### Maternal plasma 25 (OH) D concentrations by season of sampling

**Fig. 2:**
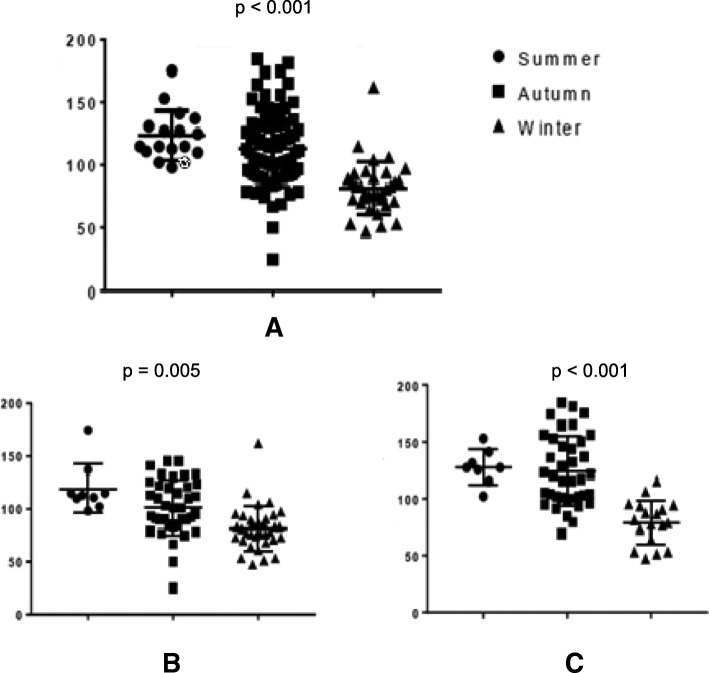
Maternal 25 (OH) D concentrations by season of sampling. **a** All participants included **b** HIV negative participants and **c** HIV positive participants

Participants were enrolled over summer, autumn and winter. We compared mean plasma vitamin D concentrations by season of enrolment (Fig. 2). Overall mean plasma 25(OH) D concentrations were significantly different by season of sampling with highest mean concentrations observed in summer. The trend was unchanged when participants were stratified by HIV status.

### Maternal 25 (OH) D and combination antiretroviral therapy (cART) duration

**Fig. 3:**
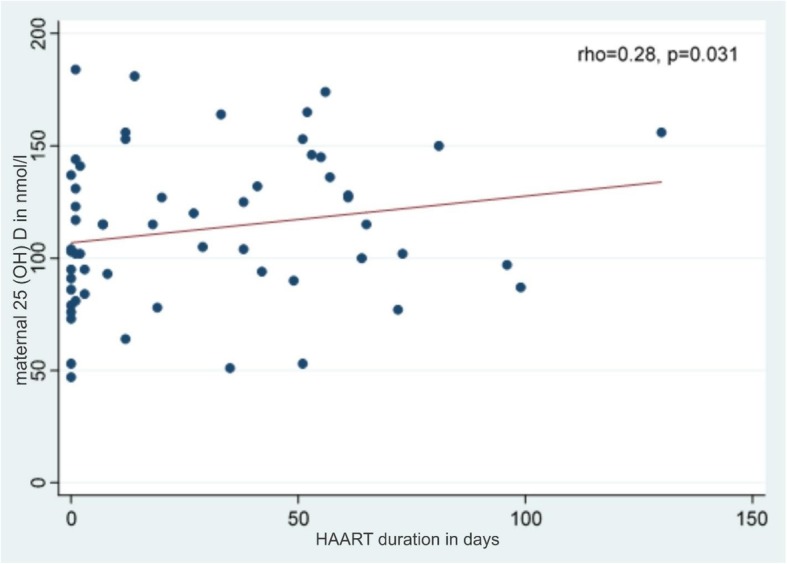
cART duration and maternal 25 (OH) D concentrations

The correlation between plasma maternal 25(OH) D and duration on cART was determined (Fig. 3). There was a weak positive correlation (r = 0.28) between maternal plasma 25 (OH) D concentrations and duration on cART.

### Determinants of maternal 25 (OH) D concentrations

HIV status and season of sampling had significant influence on maternal 25 (OH) D concentrations (Table 3). Maternal 25 (OH) D increased by 12.8 nmol/L when the HIV status changes from negative to positive. In addition the transition from summer to winter was associated with a 42.5 nmol/L decrease in 25 (OH) D (*p* <  0.001) (Table 3).

**Table 3:** Step-wise Regression of Predictible Variables

Variable	Coeffient.	Standard error	*P*-value
HIV status	12.20341	4.950488	0.015
Age	0.4354106	0.3737257	0.246
Gestational age	0.1715963	0.5217306	0.743
Season			
Autumn	−8.672066	6.71355	0.199
Winter	−44.99294	7.469283	< 0.001
Removing variables with *p* > 0.1			
HIV Status	12.80705	4.696735	0.007
Winter season	−42.48865	7.860597	< 0.001

## Discussion

### Maternal 25 (OH) D concentrations

To our knowledge, this is the first study to investigate 25 (OH) D concentrations, determinants of 25 (OH) D in late pregnancy on Zimbabwean women. The prevalence of 25 (OH) D deficiency, insufficiency and sufficiency in our study was 1.6, 11.0 and 87.4% respectively. This was an unexpected finding in that most literature cites a widespread global vitamin D deficiency in pregnant women ranging from 10 to 90% in non-African and 21–37% in African populations [25, 29–33]. Such differences could indicate that our population has higher vitamin D levels incomparison to current literature. Another possibility is that the cut-off values used in defining vitamin D deficiency in previous literature were inappropriate for pregnant women [27, 28].

Our results are however in concordance with findings on pregnant Tanzanian women which reported 1% vitamin D deficiency, 2% insufficiency and an overall mean of 138.5 (± 35.0) nmol/L [24]. In the current study, mean 25 (OH) D was 106 nmol/L (± 30.9). As expected the mean plasma vitamin D concentration was higher in these pregnant participants than the 75–84.7 nmol/L reported on non-pregnant African populations [1, 3, 4, 10].

The apparent differences between 25 (OH) D concentrations in the pregnant versus the non-pregnant could be explained by the physiology of vitamin D metabolism. During pregnancy, the concentration of vitamin D-binding protein (DBP) increases in response to elevations in oestrogen [24, 29]. The higher binding capacity of plasma 25 (OH) D and expansion of blood volume in pregnancy translates to an increased 25(OH) D pool size. In the third trimester, there is also reduced insulin sensitivity which enhances sequestration of vitamin D from the adipose tissue thereby increasing the circulating pool of 25 (OH) D [34]. Furthermore, there is a pregnancy associated decoupling mechanism on the delaying the deactivation of 25 (OH) D by the enzyme placental 24-hydroxylase (CYP24A1) thereby increasing the half-life of 25 (OH) D [35].

On the other hand there is a compelling need to establish specific reference ranges for pregnant women. Currently the Institute of Medicine and the Endocrine Society guidelines does not have 25 (OH) D reference ranges in pregnant women [36].

### Determinants of maternal 25 (OH)D

#### Maternal 25 (OH) D and HIV status

Our finding of significantly higher maternal 25 (OH) D concentrations in HIV-infected women is in discordance with findings from a number of studies that have reported an association between HIV infection and lower mean plasma values of maternal 25 (OH) D [1, 3, 4]. We speculate this difference could have been due to the effect of cART. However, the effects of cART on vitamin D are regimen dependent. Some drugs are associated with lower levels and some with higher levels. Although not assessed in this study, prolonged exposure to sunlight and improved well-being could also have contributed to the higher maternal 25 (OH) D levels.

Although information on CD4 wasn’t included in this analysis, we speculate that with prolonged use of cART, the immune status of those individuals had improved. In support, Ramayo et al reported that vitamin D deficiency was more prevalent among cART-naive patients as compared to those receiving cART (*p* = 0.04) [18]. According to Haug et al., prolonged use of cART is associated with improved 25 (OH) D concentrations along with immunological and clinical parameters of disease progression such as CD4 and tumor necrosis factor (TNF)-α [36]. HIV induced chronic inflammation characterised by TNF-α overproduction, may result in renal 1α-hydroxylase impairment [18] leading to reduced parathyroid stimulatory effect on 1,25(OH)2D production thereby leading to the accumulation of the substrate 25(OH) D in circulation.

On stratifying study participants by HIV status, a significant association was observed between cART duration and maternal 25 (OH) D concentrations (*p* = 0.031, Spearman correlation = 0.28) (Fig. 3-3).

We observed significant correlation between duration on cART and maternal plasma 25(OH) D concentrations, a finding that is conflicted with reports in Caucasian populations that reported that cART therapy particularly NNRTIs as risk factors that further decreased 25 (OH) D levels [18, 37]. This was not the case in our population, which could be attributed to racial differences, possibly genetic polymorphisms in the metabolism of vitamin D.

#### Maternal 25 (OH) D and cART

At the time of enrolment, the HIV-infected study participants were on a cART regimen of Tenolam E (Tenofovir/Efavirenz/Lamivudine). Tenofovir and Efavirenz have been reported as having direct effects on enzymes involved in vitamin D metabolism, with the majority of studies reporting an association of these drugs with 25 (OH) D deficiency [38, 39]. The combined effect of these drugs has not been investigated in literature therefore making it difficult to decipher the association with the unexpected elevation in 25 (OH)D. Individually, efavirenz has been shown to induce cytochrome P450 CYP24, which is responsible for adding a hydroxyl group on both 25(OH) D and 1,25(OH)2 converting both to their inactive metabolites [40]. Efavirenz also reduces the expression of the cytochrome CYP2R1, which is responsible for vitamin D activation [41]. Tenofovir exerts its effect by inducing tubular dysfunction thereby compromising the function of the kidney enzyme 1a-hydroxylase which converts 25 (OH) D to 1,25(OH) D [42]. These individual effects would thus have lowered the maternal serum 25 (OH) D concentrations in our population, which was not the case.

#### Maternal 25 (OH) D and season of sampling

As expected, summer was associated with the highest maternal plasma 25 (OH) D concentrations. This is in agreement with a number of published studies that have reported positive associations between seasonal changes and vitamin D status [43–45]. This effect is due to the seasonal variations in solar radiation, with the highest intensity occurring in summer resulting in maximal dermal synthesis of vitamin D. In addition, low temperatures and low relative humidity during winter are significant risk factors contributing factors to these variations in 25 (OH) D concentrations [45].

### Strengths and limitations

The strengths of this study include random sampling from four peri-urban study sites and sampling at different seasons to eliminate confounding effect of season on vitamin D synthesis. In addition, the HPLC method used for analysis of samples was traceable to the LC-MS reference method, participated in an external quality assurance scheme by DEQAS and the internal quality exhibited good precision and accuracy with the controls used.

## Conclusions

We present baseline data on vitamin D status in pregnant women in a black African population. In our setting, we documented high maternal vitamin D levels deficiency in the absence of vitamin D supplementation. HIV infection and season were significant covariates affecting the maternal vitamin D concentrations.

## Abbreviations

1,25(OH)2D: 1α, 25 –hydroxyvitamin D
25 (OH)D: 25 hydroxyvitamin D
cART: Combination Active Antiretroviral Therapy
CYP27A1: Cytochrome P450 27A1
CYP27B1: Cytochrome P450 27B1
CYP2R1: Cytochrome P450 2R1
DEQAS: Vitamin D External Quality Assessment Scheme
HIV: Human Immunodeficiency Virus
IFCC: International Federation of Clinical Chemistry
NIST: National Institute of Standards and Technology SRM: Standard reference material
nmol/L: nano-moles per liter
NNRTIs: Non-Nucleoside Reverse Transcriptase Inhibitors
PTH: Parathyroid Hormome
UZBCS: University of Zimbabwe Birth Cohort Study
VBP: Vitamin D binding protein
VDR: Vitamin D receptor
